# SARS-CoV-2 Rapidly Adapts in Aged BALB/c Mice and Induces Typical Pneumonia

**DOI:** 10.1128/JVI.02477-20

**Published:** 2021-05-10

**Authors:** Yufei Zhang, Kun Huang, Ting Wang, Fei Deng, Wenxiao Gong, Xianfeng Hui, Ya Zhao, Xinlin He, Chengfei Li, Qiang Zhang, Xi Chen, Changjie Lv, Xian Lin, Ying Yang, Xiaomei Sun, Zhengli Shi, Huanchun Chen, Zhong Zou, Meilin Jin

**Affiliations:** a State Key Laboratory of Agricultural Microbiology, Huazhong Agricultural University, Wuhan, Hubei, People’s Republic of China; b College of Veterinary Medicine, Huazhong Agricultural University, Wuhan, Hubei, People’s Republic of China; c Key Laboratory of Development of Veterinary Diagnostic Products, Ministry of Agriculture, Wuhan, Hubei, People’s Republic of China; d CAS Key Laboratory of Special Pathogens, Wuhan Institute of Virology, Center for Biosafety Mega-Science, Chinese Academy of Sciences, Wuhan, Hubei, People’s Republic of China; e State Key Laboratory of Virology and National Virus Resource Center, Wuhan Institute of Virology, Chinese Academy of Sciences, Wuhan, Hubei, People’s Republic of China; Loyola University Chicago

**Keywords:** SARS-CoV-2, aged BALB/c mouse model, pneumonia, COVID-19, rapid adaptation

## Abstract

Age is a risk factor for coronavirus disease 2019 (COVID-19)-associated morbidity and mortality in humans; hence, in this study, we compared the course of severe acute respiratory syndrome coronavirus 2 (SARS-CoV-2) infection in young and aged BALB/c mice. We found that SARS-CoV-2 isolates replicated in the respiratory tracts of 12-month-old (aged) mice and caused pathological features of pneumonia upon intranasal infection. In contrast, rapid viral clearance was observed 5 days following infection in 2-month-old (young) mice with no evidence of pathological changes in the lungs. Infection with SARS-CoV-2 elicited significantly upregulated production of cytokines, especially interleukin 6 and interferon gamma, in aged mice, whereas this response was much weaker in young mice. Subsequent challenge of infected aged BALB/c mice with SARS-CoV-2 resulted in neutralized antibody responses, a significantly reduced viral burden in the lungs, and inflammation mitigation. Deep sequencing showed a panel of mutations potentially associated with the enhanced infection in aged BALB/c mice, such as the Q498H mutation, which is located at the receptor-binding domain (RBD) of the spike (S) protein. We further found that the isolates cannot only multiply in the respiratory tract of mice, but also cause disease in aged mice. Overall, viral replication and rapid adaption in aged BALB/c mice were associated with pneumonia, confirming that the age-related susceptibility to SARS-CoV-2 in mice resembled that in humans.

**IMPORTANCE** Aged BALB/c mice are in use as a model of disease caused by SARS-CoV-2. Our research demonstrated SARS-CoV-2 can rapidly adapt in aged BALB/c mice through causing mutations at the RBD of the S protein. Moreover, SARS-CoV-2-infected aged BALB/c mice indicated that alveolar damage, interstitial pneumonia, and inflammatory immune responses were similar to the clinical manifestations of human infections. Therefore, our aged BALB/c challenge model will be useful for further understanding the pathogenesis of SARS-CoV-2 and for testing vaccines and antiviral agents.

## Abstract (precis)

Aged BALB/c mice are in use as a model of disease caused by SARS-CoV-2. Our research demonstrated SARS-CoV-2 can rapidly adapt in aged BALB/c mice through causing mutations at the RBD of the S protein.

## INTRODUCTION

A novel coronavirus, severe acute respiratory syndrome coronavirus 2 (SARS-CoV-2), was identified after the initial report of an acute respiratory syndrome outbreak in Wuhan, China in December 2019 (1). The World Health Organization confirmed that by late January 2021, the total number of infections worldwide was 103,201,340, including 2,237,636 deaths worldwide (https://www.who.int/emergencies/diseases/novel-coronavirus-2019). The disease was named coronavirus disease 2019 (COVID-19), and is characterized by fever, cough, and dyspnea that can progress rapidly to respiratory and cardiac failure requiring mechanical ventilation (1, 2). Despite the severity of the disease and high fatality rates, vaccines and antiviral agents to combat SARS-CoV-2 are not yet available. Appropriate animal models of this highly pathogenic virus are required to study and evaluate antiviral agents and vaccines developed.

An ideal animal model should reflect the clinical signs, viral replication, and pathology of SARS-CoV-2 infection in humans. Several animal species support SARS-CoV-2 replication and have been used as models to evaluate treatment responses and candidate vaccines. Clinical signs, viral replication, and pathology are species-dependent when SARS-CoV-2 is delivered into the respiratory tracts of mice, hamsters, ferrets, cats, and nonhuman primates (3–13). However, cost, limited availability, and individual variations among these animal models imply that the sample size is insufficient for statistical evaluation and large-scale studies are required to draw robust conclusions.

Similar to the SARS outbreak of 2002 to 2003, the severity of COVID-19 is associated with more advanced age and/or comorbidities, although the development of severe disease is not only limited to these risk groups (14–17). The heightened susceptibility of the elderly population to severe SARS-CoV-2 suggested that aged mice might also be more susceptible to SARS-CoV-2 than young mice. Therefore, in this study, we found that 12-month-old BALB/c mice have delayed viral clearance, that SARS-CoV-2 has an adaptive mutation and high-level replication, and that histopathological lesions are similar to those of humans with COVID-19, whereas the virus was rapidly cleared in young mice, with mild lung pathology. Therefore, the aged BALB/c mice can be used to establish SARS-CoV-2 mouse adaptive strains and determine the mechanism of age-dependent pathogenicity in human infection with SARS-CoV-2.

## RESULTS

### Replication and clinical response to primary infection with SARS-CoV-2.

**FIG 1:**
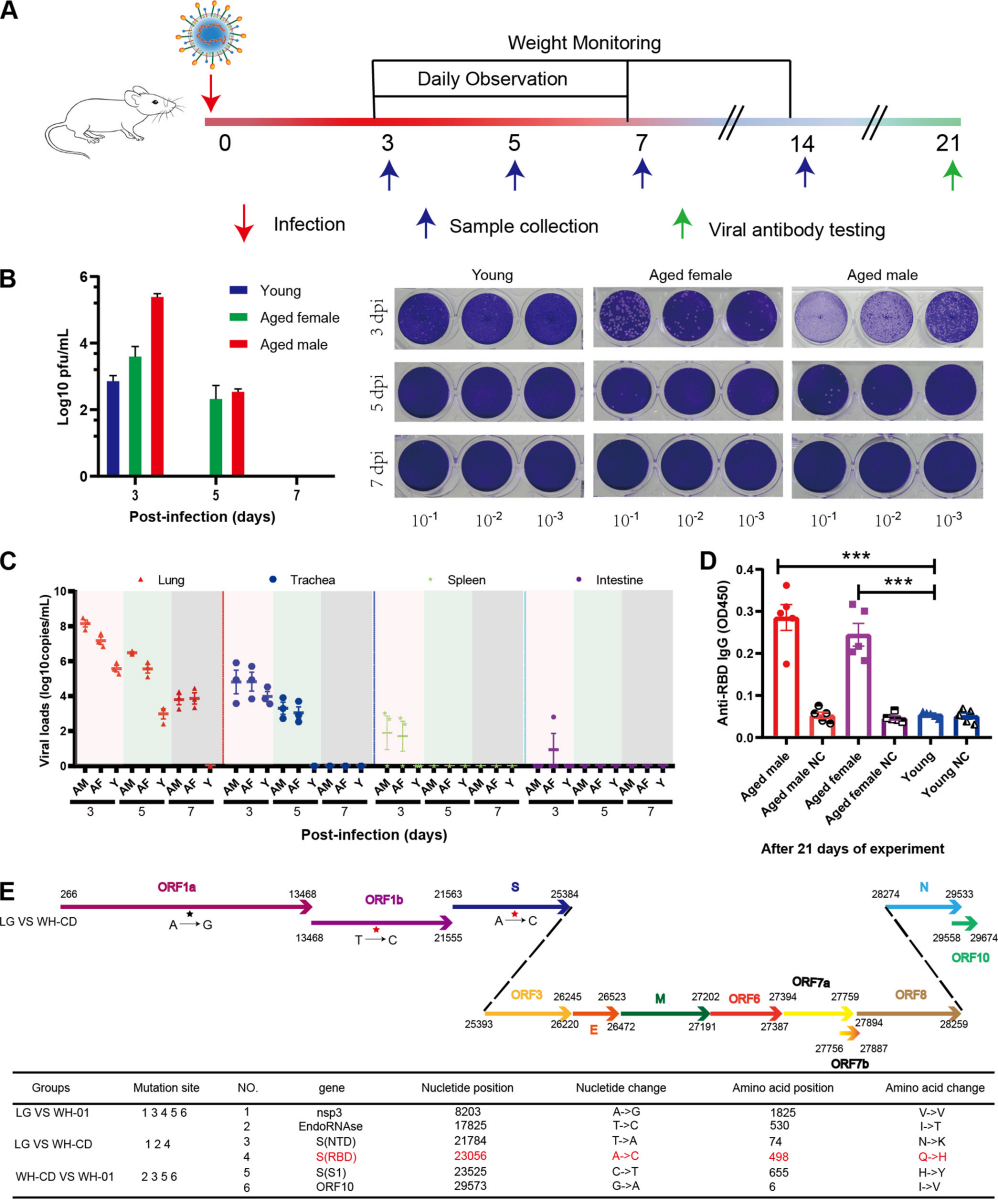
Experimental scheme, viral load, and serum antibodies in SARS-CoV-2-infected BALB/c mice. (A) Thirty-six aged BALB/c mice were intranasally infected with 1 × 10^5^ TCID_50_ virus and then euthanized to collect tissue and blood samples at 3, 5, 7, and 21 dpi. (B) Virus titers in lungs of SARS-CoV-2-challenged BALB/c mice (*n* = 3 each) at 3, 5, 7 dpi. (C) Distribution of SARS-CoV-2 in lungs, trachea, spleen, and intestines of organs of mice determined using qRT-PCR. Viral loads and titers were also assessed in these organs at 3, 5, and 7 dpi. (D) Serum-specific IgG against SARS-CoV-2 in the sera of aged male, aged female, and young mice (*n* = 5 each) at 21 dpi determined using enzyme-linked immunosorbent assay (ELISA). Data are shown as mean ± SEM; ***, *P* ≤ 0.0001. (E) Genomic architecture of SARS-CoV-2 isolated from infected mouse lungs. Schematic of SARS-CoV-2 genome, indicating mutations identified in SARS-CoV-2 isolated from mouse lungs. IgG, immunoglobulin; dpi, days postinfection; qRT-PCR, quantitative real-time reverse transcription PCR; SARS-CoV-2, severe acute respiratory syndrome-related coronavirus 2; TCID_50_, 50% tissue culture infective dose.

**FIG 2:**
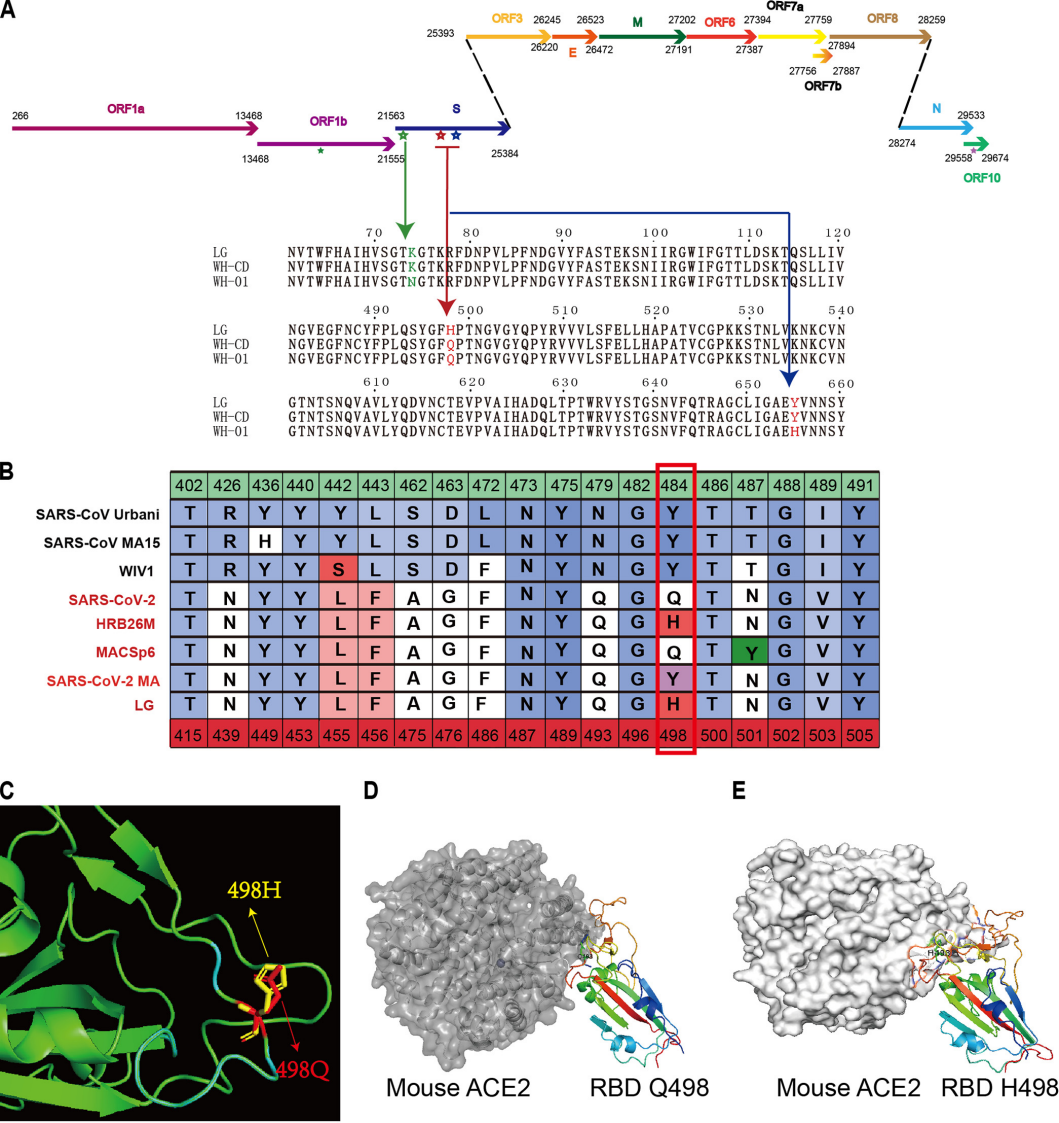
Mutations identified in the aged male mouse-isolated SARS-CoV-2 strain LG. (A) Schematic diagram of the SARS-CoV-2 genome, indicating the mutations identified in the SARS-CoV-2 strain LG, isolated from aged male mice. (B) Table of S protein RBD-ACE2-interacting residues. Amino acid positions are numbered relative to SARS-CoV (top row) and SARS-CoV-2 (bottom row). Amino acids are colored by BLOSUM62 conservation score relative to S protein from SARS-CoV Urbani (red, least conserved; blue, most conserved). SARS-CoV Urbani, SARS-CoV MA15, and WIV1 proteins can use mouse ACE2 as a functional receptor, whereas SARS-CoV-2 S cannot. The red outline indicates Q498 in SARS-CoV-2 S, which is in contact with human ACE2 in both SARS-CoV S and SARS-CoV-2 S and is divergent in SARS-CoV-2. LG isolated in infected aged mice acquired Q498H in RBD. (C) RBD in the spike structure is shown as a green helix. (D) Modeling of SARS-CoV-2 S RBD and mouse ACE2. (E) Modeling of LG S RBD and mouse ACE2.

The aged and young BALB/c mice were intranasally infected with 1 × 10^5^ the 50% tissue culture infective dose (TCID_50_) of the virus (WH-CD) and euthanized to collect tissue and blood samples at 3, 5, and 7 days postinfection (dpi) (Fig. 1A). Nine mock-infected aged male and female BALB/c mice served as controls. Following intranasal inoculation, viral RNA replication determined using quantitative real-time reverse transcriptase PCR (qRT-PCR) was robust through 3 and 5 dpi in the lungs of these BALB/c mice, with peak viral RNA loads of ∼10^10^ copies/g at 3 dpi. Infectious SARS-CoV-2 was salvaged from lung samples at 3 and 5 dpi using plaque assays. Nevertheless, no infectious virus was detectable at 7 dpi despite the persistently high numbers of viral RNA copies (Fig. 1B and C). Infectious virus was undetectable in the brain, heart, duodenum, liver, spleen, and kidneys, although a few viral RNA copies were detected at 3 and 5 dpi in the duodenum and spleen (Fig. 1C). The virus was also detected in the respiratory organs of the younger mice at 3 dpi, but not in any other organs tested. However, the infection was cleared by day 5. Body weight and clinical signs were recorded daily throughout the 14-day experiment. No clinical signs were visible during the initial 2 weeks of infection. All mice survived to the endpoint without evident symptoms or significant decreases in body weight (data not shown). Blood samples were collected at 21 dpi from mice inoculated with SARS-CoV-2, and specific antibodies were assayed in sera using ELISA. Specific IgG antibodies against the receptor-binding domain protein of SARS-CoV-2 were detected in aged mouse serum at 21 dpi (Fig. 1D). In contrast, none of the young or control mice produced antibodies against SARS-CoV-2. Subsequently, we attempted to isolate SARS-CoV-2 from the infected aged mice. Inoculating Vero E6 cells with supernatants of infected aged mouse lung homogenates caused a cytopathic effect (CPE) in the infected cells (data not shown) compared with the control. In comparison with the genome of WH-01 (Fig. 1E, bottom), the LG strain isolated from the lungs of aged mice had the following nucleotide mutations: (i) one synonymous variant, A8203G (nsp3, V-V); and (ii) the remaining mutations that were nonsynonymous, including T21784A (spike, NTD, N74K), A23056C (spike, RBD, Q498H), C23525T (spike, S1, H655Y), and G29573A (ORF10, I6V). In contrast to the genome of WH-DC (Fig. 1E, bottom), the LG had the following nucleotide mutations: (i) one synonymous variant, A8203G (nsp3, V-V); and (ii) the remaining mutations that were nonsynonymous, including T17825C (EndoRNAse, I-T) and A23056C (spike, RBD, Q498H). In comparison with the genome of WH-01 (Fig. 1E, bottom, Fig. 2A), the passaged virus WH-DC had the following nonsynonymous mutations: C17825T (EndoRNAse, T-I), T21784A (spike, NTD, N74K), C23525T (spike, S1, H655Y), and G29573A (ORF10, I6V). The mutation in the S gene occurred at the RBD region, thereby changing residue 498 of the S protein from glutamine to histidine (Q498H). SARS-CoV-2 binds its receptor ACE2 through the receptor-binding domain (RBD), and residue Q498 in the RBD is one of the five key residues responsible for receptor recognition and the host range of SARS-CoV-2 (Fig. 2B) (18–21). Protein structure and affinity prediction analysis indicated the Q498H mutation could increase the binding affinity between mouse ACE2 (mACE2) and the RBD of the SARS-CoV-2 S protein (Fig. 2C). This suggests that susceptibility to intranasal infection with SARS-CoV-2 was higher among aged than young BALB/c mice. Importantly, SARS-CoV-2 effectively replicated in the respiratory tracts of all aged BALB/c mice, which might be attributed to the enhanced binding affinity between its Q498H mutant RBD and the mouse ACE2 receptor.

### Infection with SARS-CoV-2 induced the pathological features of pneumonia in infected mice.

**FIG 3:**
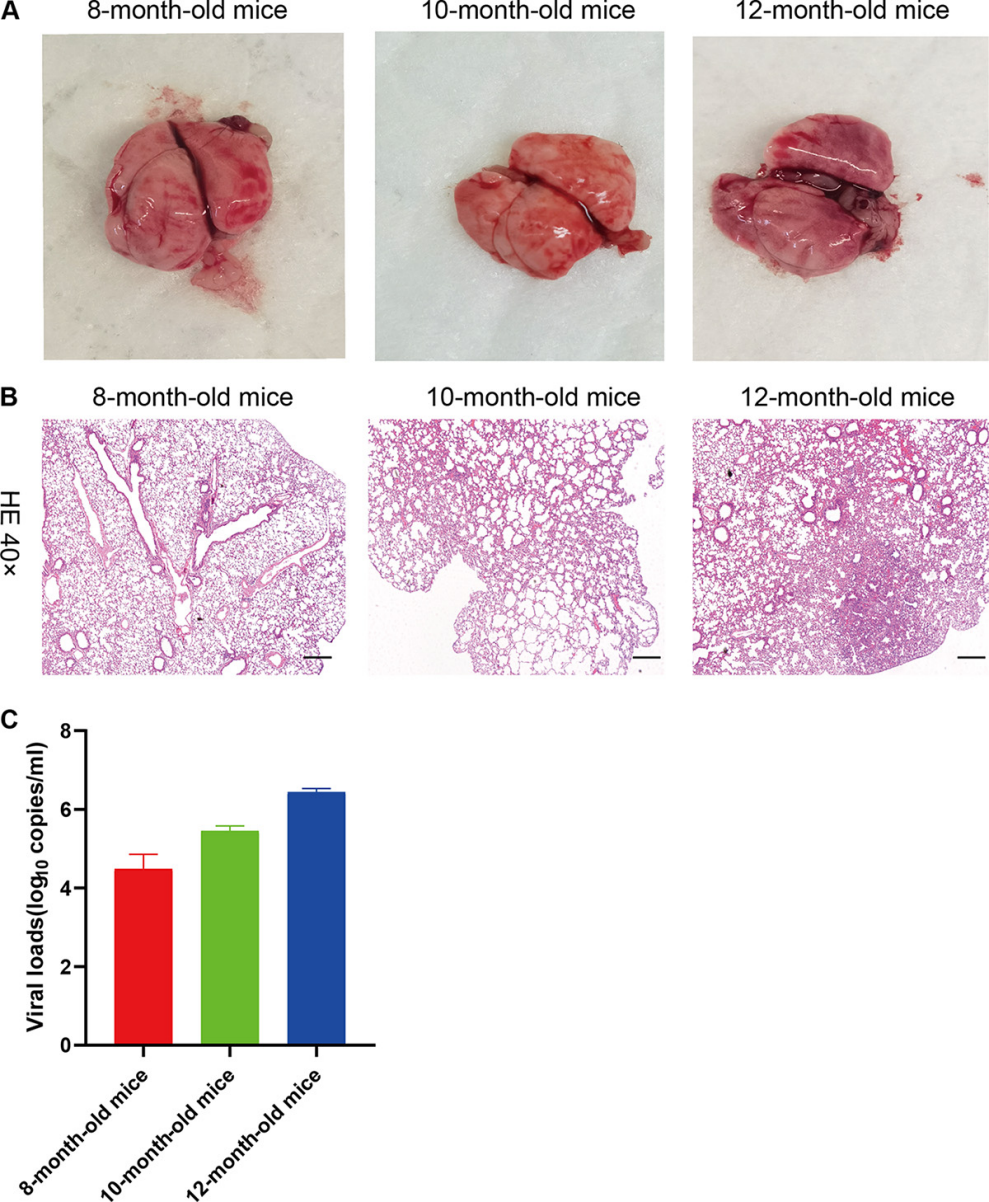
The histopathological observation of the organs in the 8-month-old, 10-month-old, and 12-month-old BALB/c mice. (A) Macroscopic appearance of lung tissue of SARS-CoV-2-infected BALB/c mice (8-month-old, 10-month-old, and 12-month-old mice) on the fifth day after infection. (B) Haemotoxylin and eosin (H&E) staining of the lungs of SARS-CoV-2-challenged BALB/c mice on 5 dpi. Eight-month-old BALB/c mice and 10-month-old BALB/c mice lacked lesions. In 12-month-old mice, H&E staining analysis shows inflammatory cell infiltration and moderate interstitial pneumonia with thickened alveolar septa. Black scale bar = 200 μm. (C) The distribution of SARS-CoV-2 in the primary organs of mice was detected using quantitative real-time PCR. These mice lungs were harvested for viral loads at 5 dpi.

**FIG 4:**
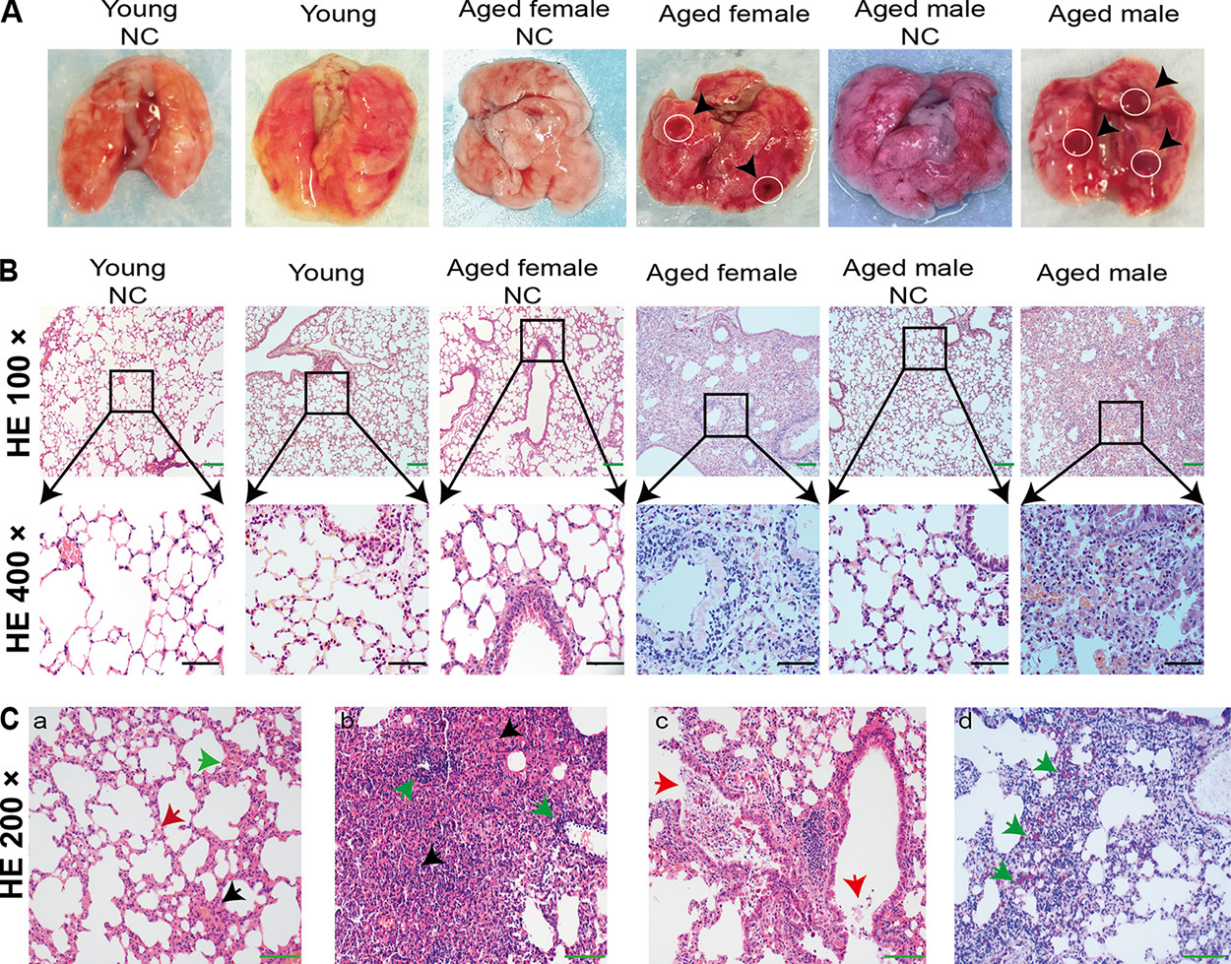
Gross pathology and histopathology of BALB/c mouse lungs after SARS-CoV-2 infection. (A) Macroscopic appearance of lung tissues from SARS-CoV-2-infected young, aged female, and aged male BALB/c mice at 5 dpi. Postmortem findings show focal dark red lesions (arrowheads). (B) Lungs of BALB/c mice challenged with SARS-CoV-2 at 5 dpi stained with H&E. Lesions are absent in mock-infected and young BALB/c mice. Staining with H&E shows inflammatory cell infiltration, and moderate interstitial pneumonia with thickened alveolar septa in aged female and aged male BALB/c mice. Black and green bars, 50 and 100 μm, respectively. (C) Multifocal lesions with lymphocyte and macrophage infiltration (a, black arrow), thickened alveolar septa (a, green arrow), and fibrin exudation (a, red arrow) in lungs from aged male BALB/c mice lung at 3 dpi. Infected aged male BALB/c mice at 5 dpi (b) had severe pneumonia with blocked terminal bronchioles, fibroplasia, and organization (b, black arrows), and peribronchial and perivascular infiltration (b, green arrows). Neutrophils, erythrocytes, fibrin, and cell debris in bronchiolar lumen (c, red arrows). Staining with MSB shows large amounts of red fibrin exudation in the alveolar septum (d, green arrows). Green bar, 100 μm. H&E, hematoxylin and eosin; dpi, days postinfection; MSB, Martius scarlet blue; NC, noninfected control; SARS-CoV-2, severe acute respiratory syndrome-related coronavirus 2.

Histopathology illustrated clear age-dependent differences (Fig. 3). Gross and histopathological changes were not evident in any of the mice at 3 dpi. By 5 dpi, all aged male mice had comparable gross lesions with focal to multifocal dark red discoloration in lung lobes, while one aged female mouse had similar histopathological changes with a slightly deflated lung lobe, compared to young or mock-infected mice (Fig. 4A). To further determine whether these mice had pneumonia, lung tissues were collected at 3 or 5 dpi, and analyzed via histopathological hematoxylin and eosin (H&E) staining. Microscopy showed moderate interstitial pneumonia characterized by thickened alveolar septa in lung tissues from aged mice at 5 dpi, but not in those of young and control mice (Fig. 4B). Furthermore, pathological sections showed different levels of pneumonia throughout the infection course. At 3 dpi, some minor changes in lung tissues from three aged male mice comprised multifocal lesions, a few thickened alveolar walls with monocyte and lymphocyte infiltration, and abundant macrophages and lymphocytes in some alveolar spaces with minor fibrin exudation (Fig. 4C, panel a) compared with control lung tissues. At 5 dpi, moderate interstitial pneumonia with thickened alveolar septa and 40 to 60% inflammatory cell infiltration was identified in the lung tissues of one aged female and three aged male mice (Fig. 4C, panel b). The aged male mice had more mixed inflammatory cell infiltration, fibrosis or histology, and hyperemia. Furthermore, lung histopathology showed increased immune infiltration and cell debris in the alveolar wall, bronchial epithelium, and bronchial lumen in SARS-CoV-2-infected aged male mice in contrast to mock-infected mice at 5 dpi. These findings indicated that SARS-CoV-2 infection induced acute bronchiolitis (Fig. 4C, panel c). The thickened alveolar septum contained macrophages, lymphocytes and neutrophils (Fig. 4C, panel d). However, the histopathological findings showed that the myocardium, liver, spleen, kidneys, cerebrum, intestine, and testes were not significantly altered (data not shown). These data further indicated that SARS-CoV-2 can infect and induce pneumonia in aged BALB/c mice and that the histological features of the lungs were very similar to those in patients with COVID-19.

### SARS-CoV-2 replication and cytopathic effects in bronchial epithelium.

**FIG 5:**
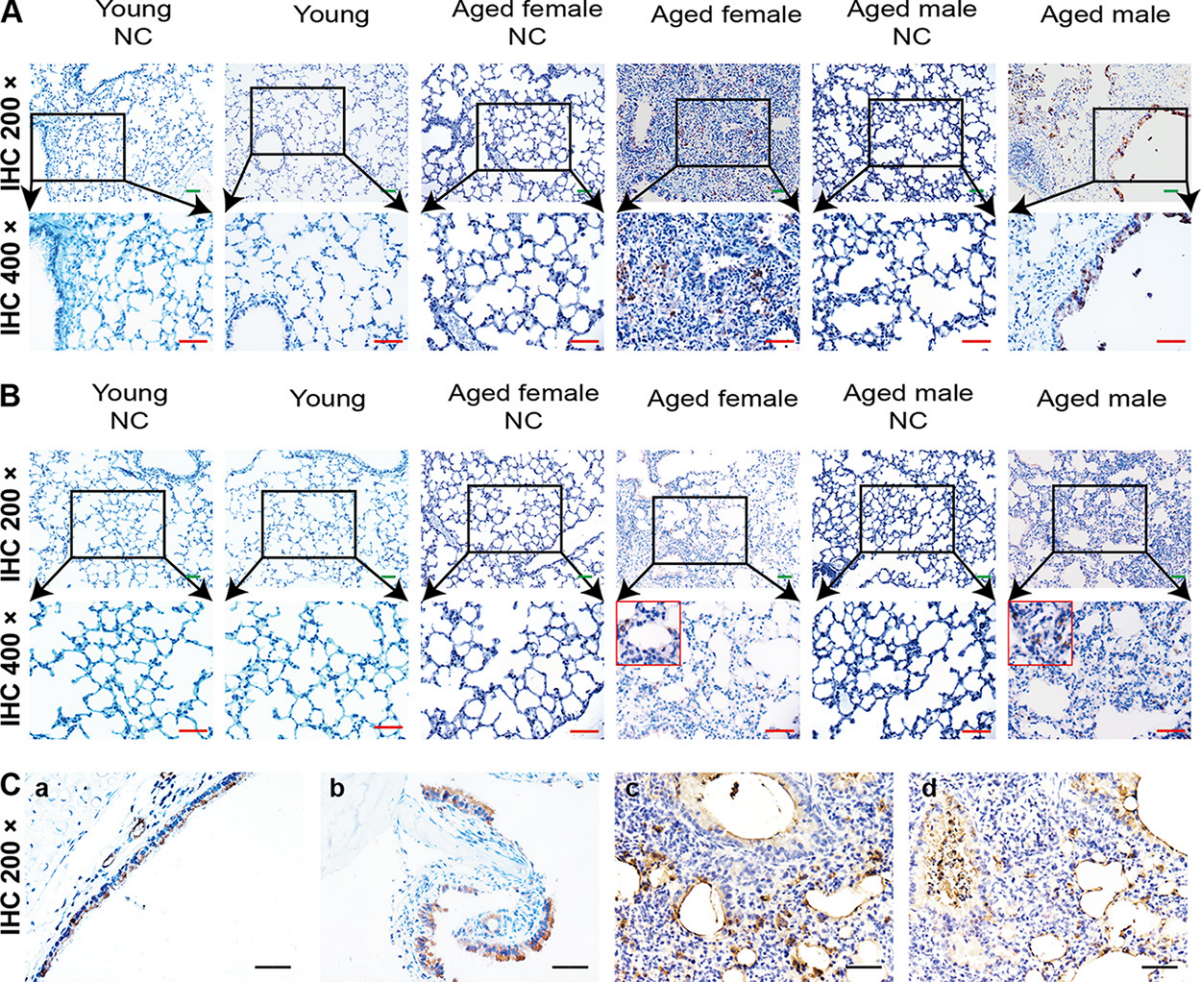
Viral antigen detection in BALB/c mice infected with SARS-CoV-2. (A) Immunohistochemical detection of viral antigen using anti-nucleocapsid-protein rabbit monoclonal antibody in lung bronchi and alveoli at 3 dpi. (B) Viral antigen detected using anti-spike-S1 rabbit monoclonal antibody in lung bronchi and alveoli at 3 dpi. (C) Tissues of SARS-CoV-2-infected BALB/c mice: (a) trachea, (b) nasal turbinate, (c and d) lungs. SARS-CoV-2 antigens in tracheal epithelial cells at 3 dpi and debris with desquamated epithelial and inflammatory cells in lumen. SARS-CoV-2 antigens at 3 dpi in epithelial surface lining of nasal turbinate, and necrotic debris in nasal passages. Viral antigens in mononuclear cells, alveolar epithelia, and degenerative and desquamating bronchial epithelial cells. Green bar, 100 μm; red bar, 50 μm; black bar, 100 μm.

**FIG 6:**
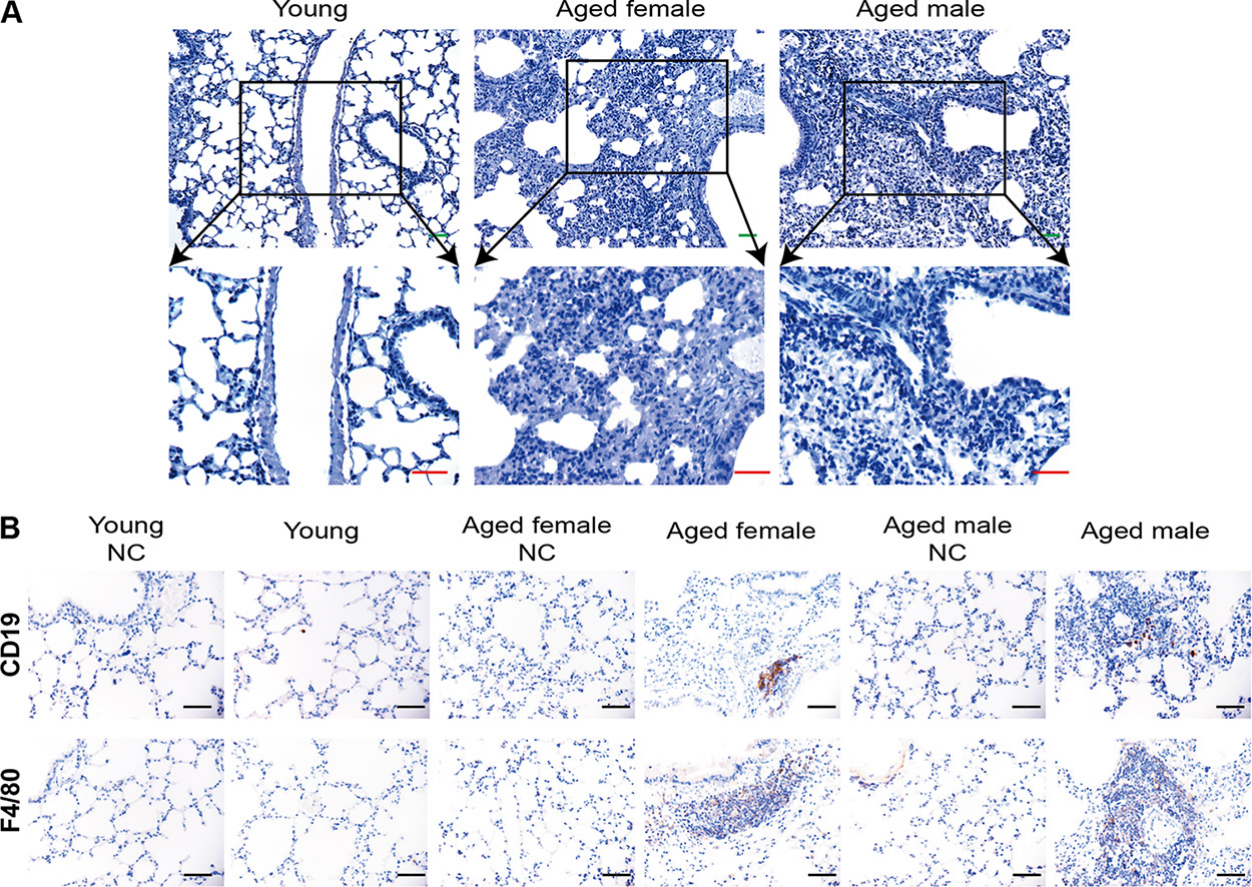
Macrophages with F4/80 and B lymphocytes with CD19 identified using IHC staining. (A) Secondary antibody-only control for both young and aged tissues. (B, top) Several B lymphocytes infiltrated the thickened alveolar septa at 5 dpi in the aged male and female BALB/c mice. (B, bottom) Diffuse infiltration of macrophages in the expanded alveolar septa in the aged male and female BLAB/c mice at 5 dpi. Secondary antibody-only control for the lung of mice.

We further confirmed viral replication in infected aged BALB/c mice using immunohistochemistry (IHC). Cells in the bronchiolar ciliated epithelial cells and alveolar epithelial cells were positive for SARS-CoV-2 N (nucleocapsid protein) (Fig. 5A) and S (spike glycoprotein) antigens (Fig. 5B) in infected aged mice, but not in mock-infected control or young mice. Additionally, negative controls (no primary antibody) were also included, and all were negative for staining (Fig. 6A). Viral antigens were evident in nasal turbinate (Fig. 5C, panel b) and tracheal (Fig. 5C, panel a) epithelia at 3 dpi. In the lesional areas of the lungs, sequential IHC-stained sections revealed viral antigens in macrophages, alveolar epithelia (Fig. 5C, panel c), and degenerative and desquamating bronchial epithelial cells (Fig. 5C, panel d). At 5 dpi, IHC staining detected less viral antigen content compared with that at 3 dpi (data not shown). Viral antigens were undetectable in nasal mucosa, turbinates, trachea, and lungs at 7 dpi (data not shown) or in the myocardium, liver, spleen, kidney, cerebrum, intestine, and testis at any time.

We assessed the infiltration of specific inflammatory cells via IHC staining to identify F4/80^+^ macrophages and CD19^+^ B lymphocytes (Fig. 6B). More macrophages and T lymphocytes accumulated in the lungs of aged mice, along with prolonged infection, compared with young mice. F4/80^+^ macrophages diffusely infiltrated the alveolar cavities at 3 dpi, and focally aggregated in thickened alveolar septa at 5 dpi. CD19^+^ B lymphocytes were dispersed in the alveolar interstitium in the aged mice at 5 dpi. These results indicated that SARS-CoV-2 can replicate in the respiratory tract of wild type aged BALB/c mice, resulting in more severe inflammatory cell infiltration.

### Kinetic analysis of inflammatory serum cytokine levels in SARS-CoV-2-infected mice.

**FIG 7:**
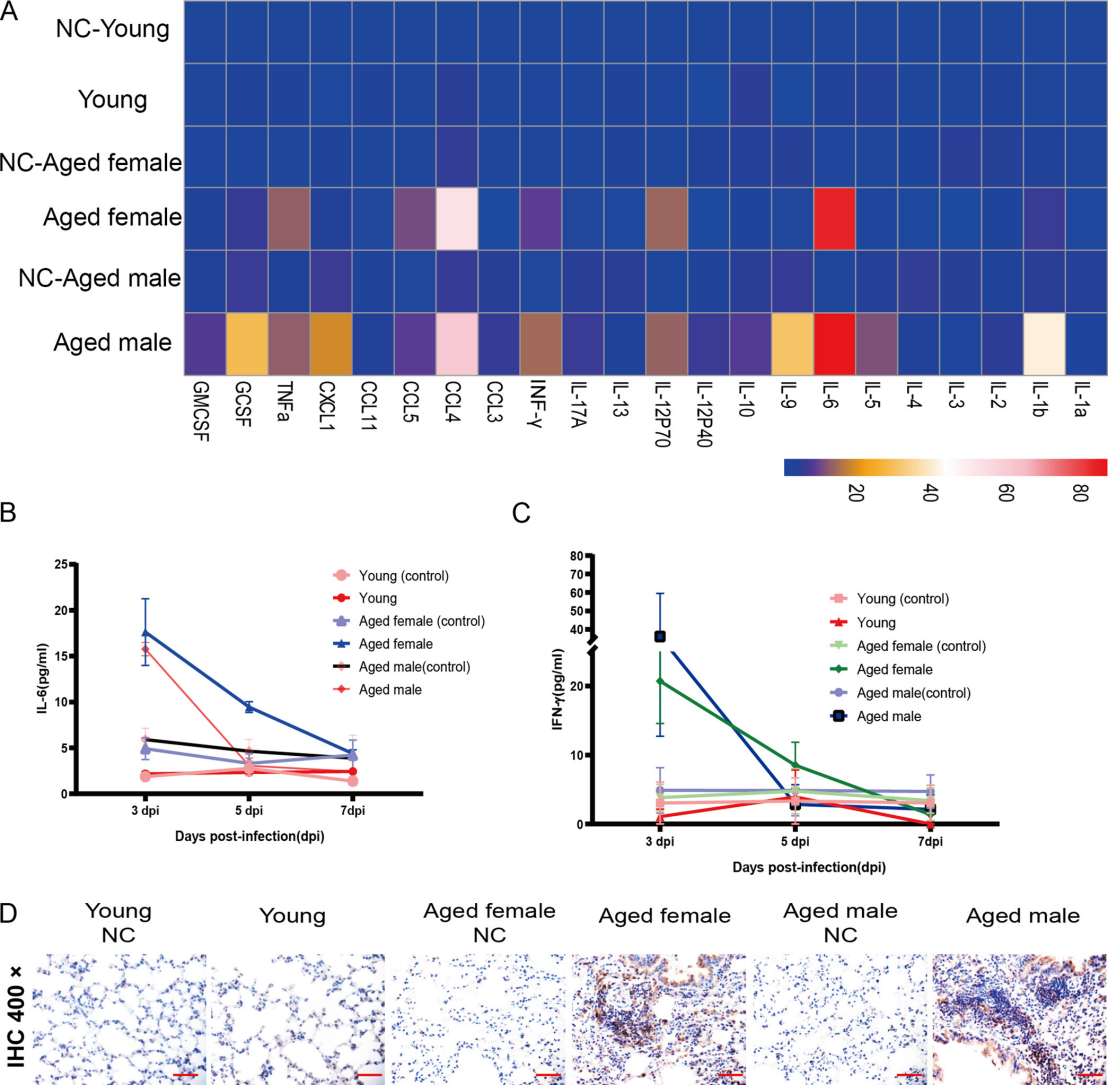
Kinetic analysis of serum inflammatory cytokines in BALB/c mice infected with SARS-CoV-2. (A) Lung cytokine and chemokine heatmap in SARS-CoV-2-infected young and aged mice. Data are presented as fold change relative to young mice mock infected with SARS-CoV-2 (*n* = 3). (B and C) Concentrations of IL-6 (B) and IFN-γ (C) in serum at different time points in aged female, aged male, and young BALB/c mice infected with SARS-CoV-2, and negative control mice. (D) Immunohistochemical staining of lung tissues from BALB/c mice infected with SARS-CoV-2 shows IL-6 expression in inflammatory lymphocytes and monocytes at sites of peribronchial and perivascular infiltration and few alveolar epithelial cells. Black bar, 50 μm. IFN-γ, interferon gamma; IL-6, interleukin 6; SARS-CoV-2, severe acute respiratory syndrome-related coronavirus 2.

An excessive proinflammatory host response to SARS-CoV-2 infection contributes to pulmonary pathology and the development of respiratory distress in some COVID-19 patients (17, 22, 23). To compare the host responses in SARS-CoV-2-infected young and aged mice, we performed qRT-PCR on lung homogenates at 3 dpi. As shown in Fig. 7A, SARS-CoV-2 infection led to elevated cytokine mRNA levels, including tumor necrosis factor α (TNF-α), chemokine (C-C motif) ligand 5 (CCL5), CCL4, gamma interferon (IFN-γ), interleukin 6 (IL-6), and IL-12-P40 in aged BALB/c mice, but it had a weaker response in young mice. Conversely, serum levels of inflammatory cytokines, such as IL-2, IL-7, IL-10, and TNF-α, were changed in patients with COVID-19 (23, 24). Therefore, we investigated kinetic changes in serum levels of IL-2, IL-4, IL-6, IL-17A, IL-10, IFN-γ, and TNF-α in mice infected with SARS-CoV-2. These cytokines changed like those of patients with COVID-19, and IL-6 and IFN-γ were significantly upregulated upon SARS-CoV-2 challenge. The serum cytokine response observed in young mice was much milder in the aged mice following SARS-CoV-2 challenge (Fig. 7B and C). Thus, the aged mice not only sustained viral replication but also developed pulmonary pathology upon SARS-CoV-2 infection. We further stained tissues using IHC to determine the contribution of locally produced IL-6 to the inflammatory component of lung damage. The expression of local IL-6 immunoreactivity was increased in inflammatory cells (lymphocytes and monocytes) at sites of peribronchial and perivascular infiltration and in a few alveolar epithelial cells (Fig. 7D). Although IL-6 production by infected cells could contribute to immunopathology, local production could also be involved in macrophage recruitment and epithelial reparative processes (25–27). An increased level of IL-6 in patients is considered an important predictor of the course of most serious diseases and a need for intensive care among patients infected with COVID-19. Herein, we found significantly increased serum levels of IL-6 in aged BALB/c mice infected with SARS-CoV-2. These findings indicated that the mechanisms through which IL-6 might contribute to disease exacerbation and the potential for therapeutic approaches based on anti-IL-6 agents could be investigated in aged BALB/c mice.

### Previously exposed aged BALB/c mice resisted challenge with SARS-CoV-2.

**FIG 8:**
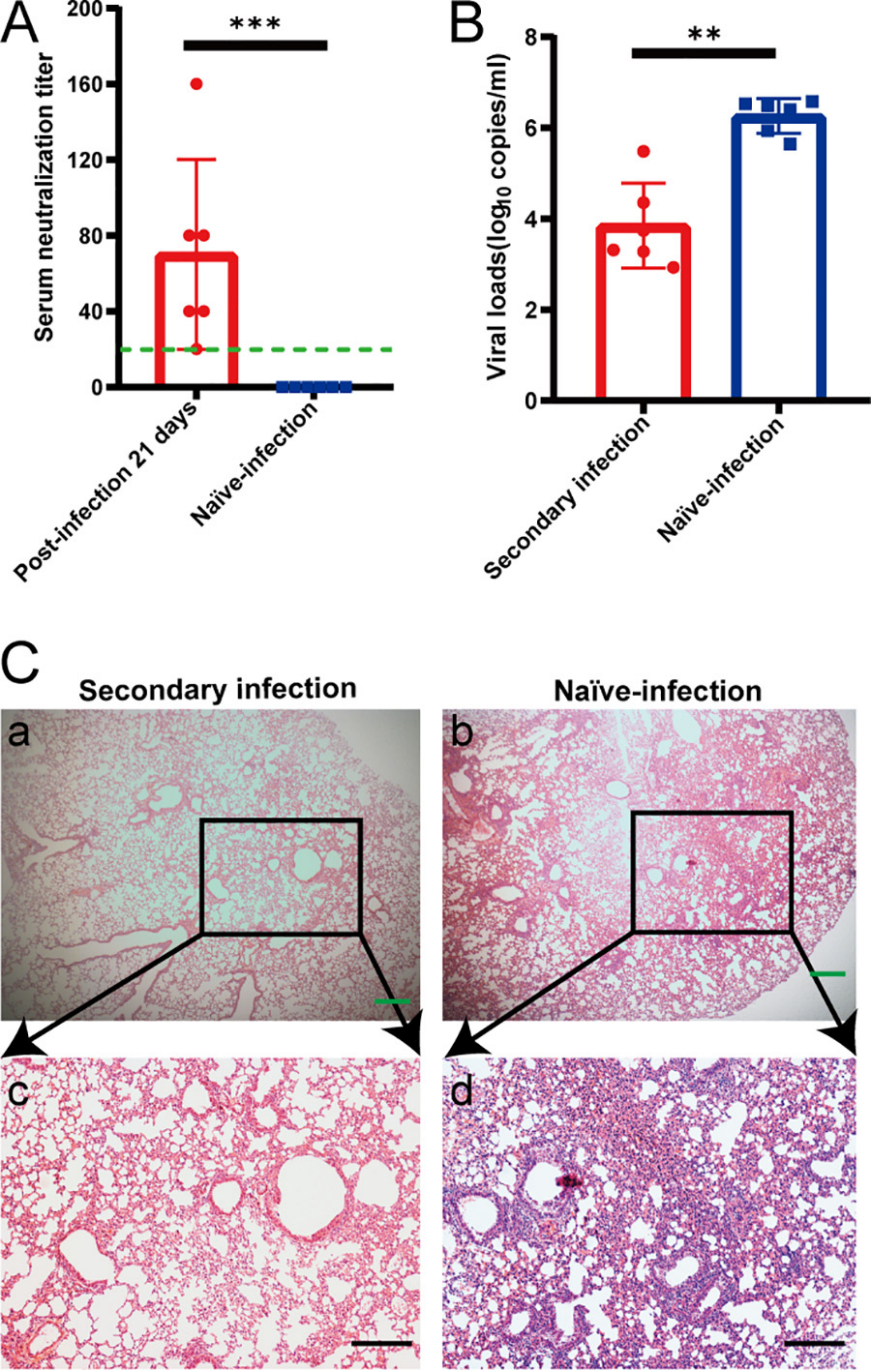
High titer of neutralizing antibodies generated during initial SARS-CoV-2 infection can protect aged mice from reinfection. Six aged mice that were either previously infected or not with SARS-CoV-2 were inoculated with 1 × 10^5^ TCID_50_ SARS-CoV-2 via nasal cavity at 21 and 5 dpi. Levels of serum neutralizing antibody and viral load in lungs were measured in euthanized mice at 5 dpi after the second inoculation. (A) Serum neutralizing antibody titers against SARS-CoV-2 were measured in Vero cells after 21 dpi (*n* = 6 per group). Dotted line, detection limit (serum neutralizing antibody titer = 1:20). Data are shown as mean ± SEM. ***, *P* ≤ 0.0001. (B) SARS-CoV-2 distribution in mouse lungs detected using qRT-PCR. Viral load in lungs of these mice were assessed at 5 dpi. Data are shown as mean ± SEM. **, *P* ≤ 0.001. (C) Previously infected mice had mild alveolar septum widening, fused pulmonary alveoli, and inflammatory cell infiltration (panels a and c). Peribronchial and perivascular infiltration in naive-infected mouse lungs (panels b and d). Green and black scale bars, 200 and 100 μm, respectively. qRT-PCR, quantitative real-time reverse transcription PCR; dpi, days postinfection; SARS-CoV-2, severe acute respiratory syndrome-related coronavirus 2; TCID_50_, 50% tissue culture infective dose.

**FIG 9:**
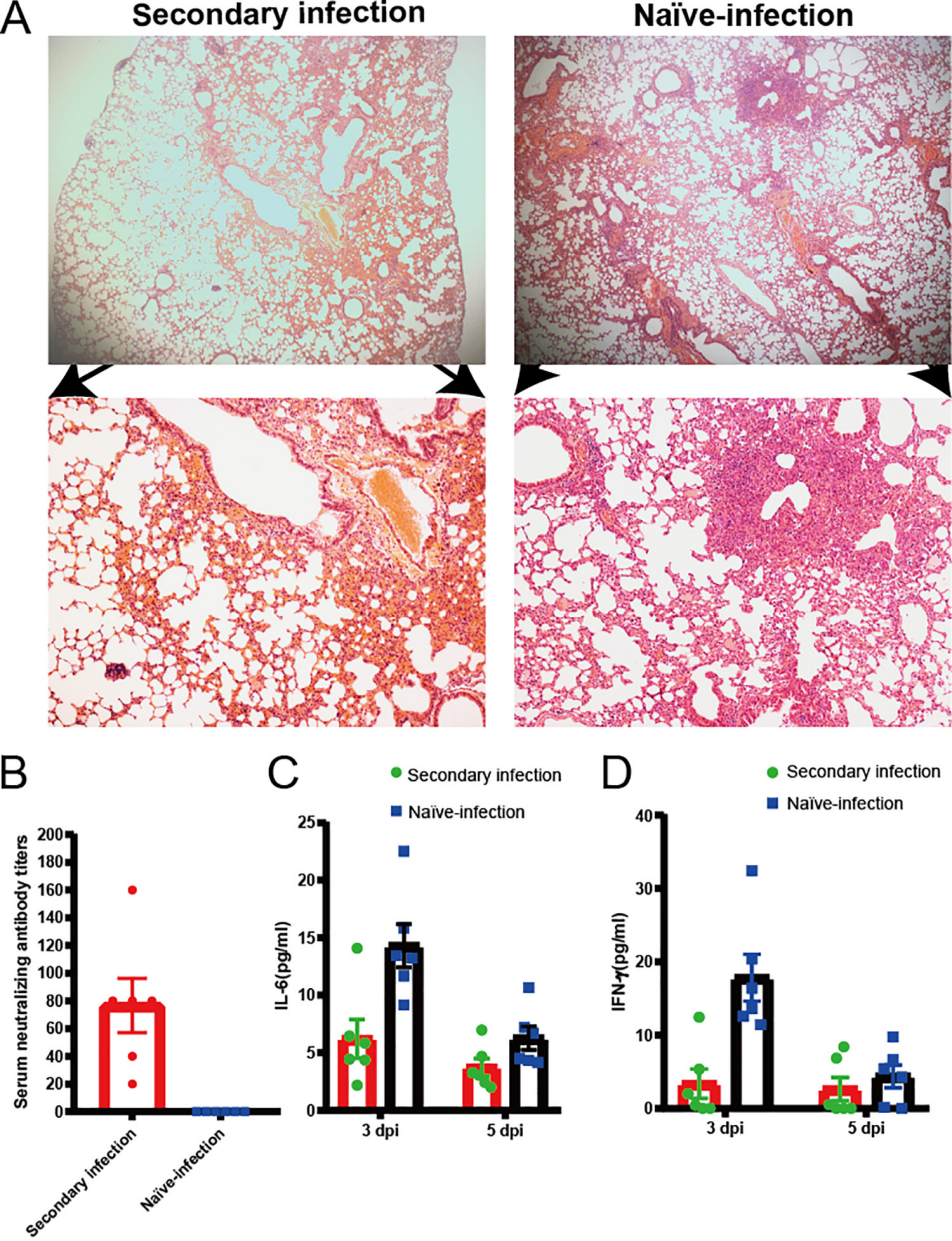
Low levels of neutralizing antibodies cannot protect mice from SARS-CoV-2 reinfection. Pathological sections of lungs from previously challenged aged mouse with neutralizing antibody titer of 1:20 (A, panels a and c) and naive-infected mouse (A, panels b and d) show peribronchial and perivascular infiltration. Green and black bars, 200 and 100 μm, respectively. (B) Serum neutralizing antibody titers against SARS-CoV-2 were measured in Vero cells after second infection 5 dpi (*n* = 6 per group). Dotted line, detection limit (serum neutralizing antibody titer = 1:10). Data are shown as mean ± SEM. (C and D) Concentrations of IL-6 (C) and IFN-γ (D) in serum at different time points in second-infected (• green) and naive-infected (■ blue) BALB/c mice infected with SARS-CoV-2.

We evaluated whether the aged BALB/c mice model can serve as a platform for testing the outcomes of therapeutics against SARS-CoV-2 infection. We infected six aged BALB/c mice with SARS-CoV-2, with identical viral doses via the route of infection described above and observed them for 21 days. These mice generated antibodies against SARS-CoV-2 that neutralized 100 TCID_50_ of the virus at dilutions from 1:20 to 1:160 (Fig. 8A). We then intranasally challenged these mice and another six aged BALB/c mice with 1 × 10^5^ TCID_50_ of virus. All mice were euthanized at 5 dpi to evaluate viral titers and pathological changes in the lungs. The lungs of challenged, aged mice contained significantly fewer viral RNA copies than those of the naive-infected mice (Fig. 8B). Histopathology of the lungs of the reinfected survivor cohort showed moderate inflammatory cell infiltration. Interestingly, pathological lung sections did not show a significant difference between an aged mouse with a neutralizing antibody titer of 1:20 and an aged mouse infected with SARS-CoV-2 for the first time (Fig. 9A). In addition, serum neutralizing antibody titers and cytokines were detected in animals after challenge (Fig. 9B to D). In contrast, peribronchial and perivascular inflammatory cell infiltration was found in the naive-infected group (Fig. 8C). These data showed that prior exposure to SARS-CoV-2 significantly reduced further lung damage in mice challenged with a second dose of SARS-CoV-2. This underscores the value of this model for future therapeutic studies.

### LG isolates cannot only multiply in the respiratory tract of mice, but also cause disease in aged mice.

**FIG 10:**
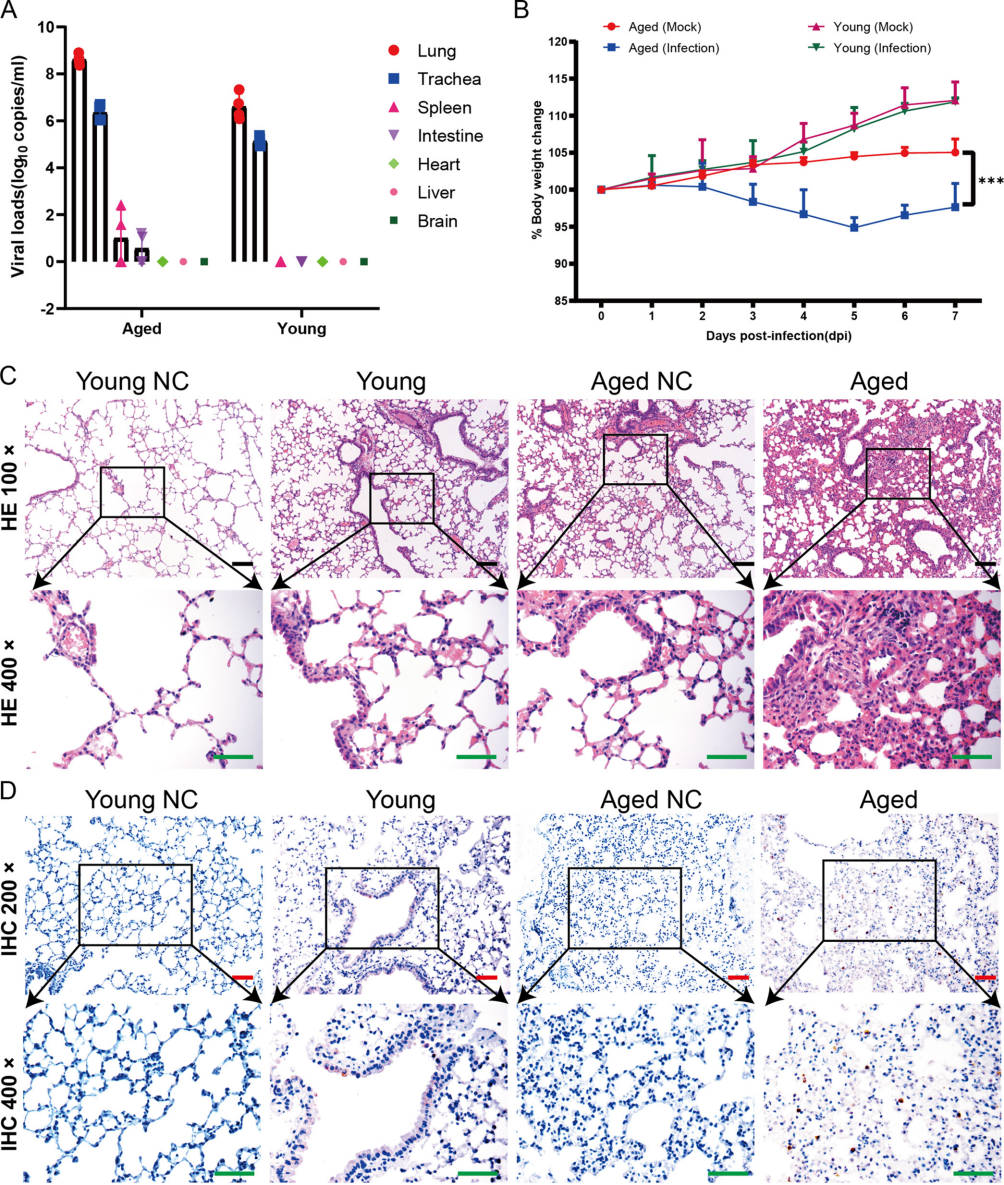
SARS-CoV-2 LG replicates in young and aged BALB/c mice. Groups of nine 6- to 8-week-old female BALB/c mice and 12-month-old male BALB/c mice were inoculated i.n. with 10^5^ TCID_50_ of LG in a volume of 50 μl. On days 3, 5, and 7 p.i., three mice were each euthanized and their tissue collected for virus detection. (A) The viral RNA copies in each organ were detected using qPCR. (B) Groups of 6- to 8-week-old (young) female BALB/c and 12-month-old female BALB/c mice were i.n. inoculated with 10^5^ TCID_50_ of LG or PBS (mock). Body weights were monitored daily for 7 days and are presented as a percentage of the weight on the day of inoculation (day 0). (C and D) Histopathologic (C) and immunohistochemical (D) studies were performed on samples from the LG-inoculated young female mice and aging adult male mice.

**FIG 11:**
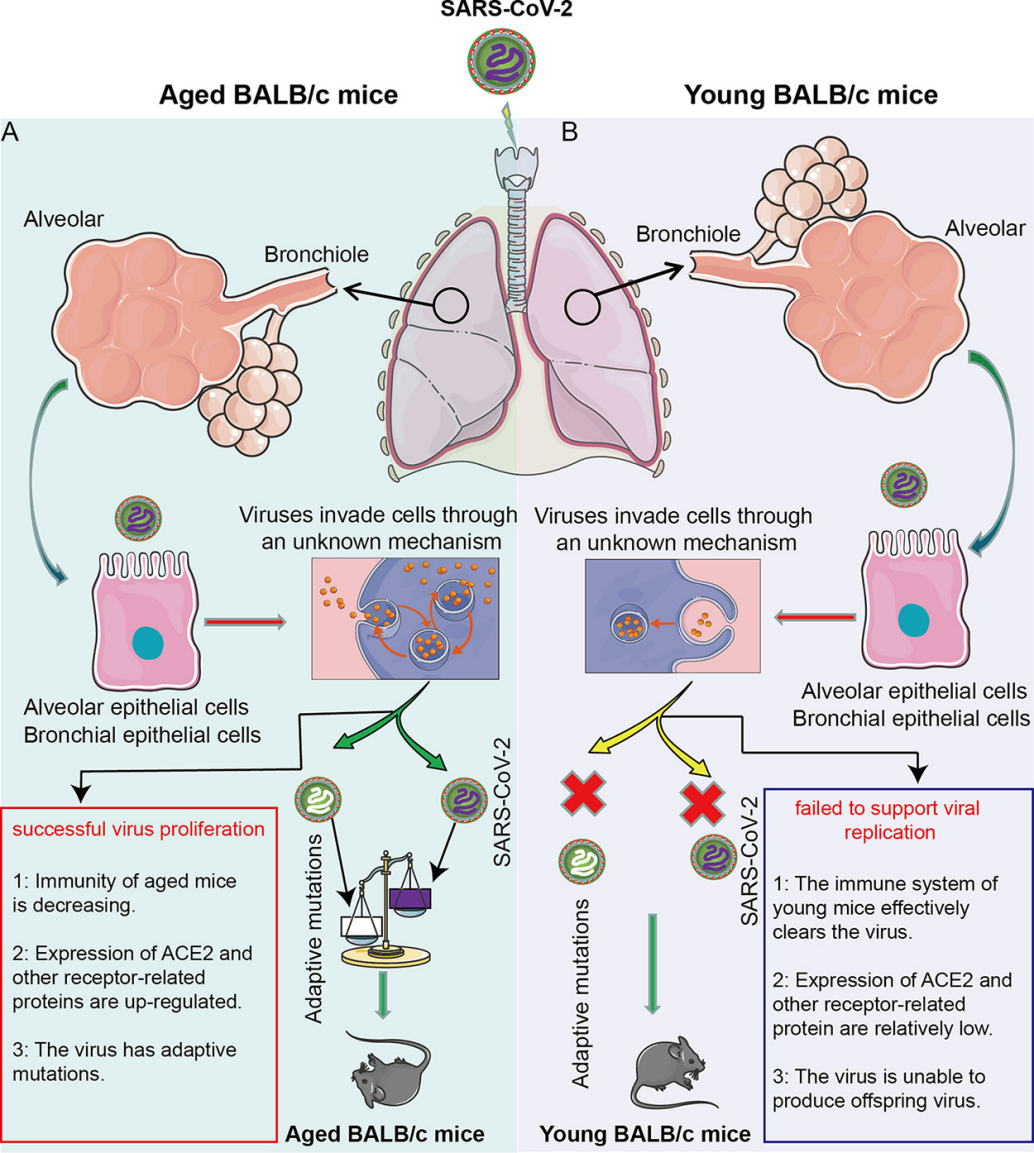
Models of the SARS-CoV-2 virus rapidly adapting in aged BALB/c mice and inducing typical pneumonia. SARS-CoV-2 passes through the mouse trachea and bronchioles to reach the alveoli. In this process, SARS-CoV-2 mainly contacts with tracheal epithelial cells, bronchiolar epithelial cells, and alveolar epithelial cells. These cells swallow SARS-CoV-2 virus particles into the cells via an unknown or nonspecific mechanism. If these cells are derived from aged BALB/c mice (A), SARS-CoV-2 will have a greater chance to proliferate and a higher chance of adaptive mutation as well. The possible reason is that the immune function of aged BALB/c mice is weakened and cannot effectively inhibit the proliferation of SARS-CoV-2. In addition, SARS-CoV-2 provides the necessary conditions for adaptive mutations in aged BALB/c mice after the proliferation. The adaptively mutated virus effectively multiplies, causing lung lesions in aged BALB/c mice. However, in cells derived from young BALB/c mice (B), SARS-CoV-2 cannot proliferate, since young BALB/c mice have an optimum immune function, which can effectively inhibit the proliferation of SARS-CoV-2. In the meantime, the probability of adaptive mutations is relatively small. Therefore, SARS-CoV-2 cannot effectively proliferate in young BALB/c mice, nor will it cause lung disease.

Although SARS-Cov-2 cannot use mice ACE2 to invade cells, the results in our research showed that SARS-Cov-2 not only infected aged BALB/c mice, but also caused their pathological changes. Applying the whole-genome sequencing method to the LG strain isolated from the lungs of aged mice, we found that the mutation in the S gene occurred at the RBD region, leading to the changes of residue 498 of S protein from glutamine to histidine (Q498H). This indicated that the pathological changes in aged mice were primarily caused by the adaptive mutant strain LG of SARS-CoV-2. To verify this, we inoculated the isolated LG strains into aged BALB/c mice and young BALB/c mice and found that LG strains could effectively proliferate in aged BALB/c mice and young BALB/c mice (Fig. 10A). In the meantime, the weight of the aged mice was slightly decreased, but that of young mice showed no evident changes (Fig. 10B). Pathological observation showed that the lung lesions of the aged BALB/c mice were obvious, but those of young mice were not, with only slight lymphocyte exudation (Fig. 10C). The results of immunohistochemistry showed significantly more SARS-CoV-2 nucleoprotein (NP) antigen-positive cells in the lungs of aged BALB/c mice than in young mice (Fig. 10D), which further suggested that aged BALB/c mice can accelerate the adaptive mutation of SARS-CoV-2. At the same time, these adaptively mutated viruses, such as the LG isolates, reinfected aged mice, causing these perceptible pathological changes (Fig. 11A). In contrast, young mice have a normal immune system to protect them against SARS-CoV-2, making it much more difficult to produce adaptive mutations and therefore cannot produce pathogenicity (Fig. 11B).

## DISCUSSION

The sudden emergence of pandemic SARS-CoV-2 has caused worldwide concern and threatened global health. The scientific community has been rigorously attempting to create vaccines or drugs that will be effective against SARS-CoV-2. Appropriate animal models are needed to explore the pathogenesis of SARS-CoV-2 and to test new antiviral therapies and vaccines. An ideal animal model should reproduce the viral replication and clinical outcomes of patients with COVID-19. We show here that SARS-CoV-2 rapidly adapted in aged BALB/c mice. Furthermore, SARS-CoV-2 not only replicated efficiently in the lungs, trachea, and nasal turbinates of aged BALB/c mice, but also caused interstitial pneumonia. That is, the clinical features of COVID-19 in humans were similar to those of these mice. Notably, infected mice produced neutralizing antibodies against SARS-CoV-2 and the virus could be salvaged from these mice.

Viral infections begins with the viral particles binding to the receptors on the host cell surface. SARS-CoV-2 uses the ACE2 receptor molecule to invade host cells. SARS-CoV-2 cannot infect wild-type laboratory mice since the viral spike protein shows relatively low affinity for the mouse ACE2 (18–21). Consequently, for SARS-CoV-2 to infect a new mouse species, the gain-of-function to bind to the mouse ACE2 is a prerequisite. Our WH-CD isolate acquired the mutations N74K and H655Y in the S protein after 4 *in vitro* passages, compared with the original SARS-CoV-2 isolate (WH-01). WH-CD showed no pathogenicity in young mice, however, high viral titers and pathological damage were detected in aged mice infected with WH-CD. The LG strain isolated from infected mice acquired the Q498H mutation in the RBD, which may have attributed to the mouse adaptation in a previous study (18). This may be due to the special internal environment in the lungs of aging mice, which warrants further investigation. In conclusion, the Q498H mutation in the RBD contributed to the different effects of WH-CD observed in older and younger mice.

Patients with COVID-19 commonly develop pneumonia with typical signs, including fever, cough, dyspnea, muscle aches, headaches, chest pain, diarrhea, hemoptysis, sputum production, rhinorrhea, nausea, vomiting, sore throat, confusion, and anorexia (1, 28). Histopathology of the lungs shows diffuse alveolar damage (29, 30). The predominant findings in patients are hyaline membranes, alveolar septal destruction, lymphocytic infiltration of the bronchi, and extensive granulocytic infiltration of the alveoli and bronchi (30, 31). Aged BALB/c mice infected with SARS-CoV-2 developed obvious interstitial pneumonia with inflammatory cell infiltration.

Advanced age is an established risk factor for poor COVID-19 outcomes. The risk for people aged ≥80 years is >20-fold higher than that of persons aged 50 to 59 years (16, 32). The underlying reason for this might be immunosenescence (age-related decline in immune function), which is a major contributor to morbidity and mortality among the geriatric population (16, 17). Young adult BALB/c mice aged 2 months infected with SARS-COV-2 did not develop either symptoms or mortality, whereas mice aged 12 months developed pneumonia. Moreover, the viral load was significantly higher in aged mice. Elevated cytokine levels correlate with increased inflammatory damage and accurately predict morbidity and mortality (17). Levels of IL-6 expression were high in SARS-COV-2-infected aged mice, but moderate in young mice (27).

In summary, we elucidated a novel mechanism, that is, the age-related susceptibility of BALB/c mice to *de novo* SARS-CoV-2 infection. Certain factors, including aging-related genes and adaptive immune dysregulation, contribute to the high severity rate of aged COVID-19 patients (33). In this study, aged mice were susceptible to SARS-CoV-2 since it produced progeny virions adapted to mice. However, in contrast to young BALB/c mice, the enhanced and prolonged replication of SARS-CoV-2 was accompanied by clinical manifestations, alveolar damage, and interstitial pneumonia in aged mice, which was similar to that of humans. The expression of proinflammatory cytokines was increased in mice infected with SARS-CoV-2, but not in aged mice with mock infection. No single animal model for SARS-CoV-2 can currently reproduce all aspects of the human manifestation of COVID-19 disease. Therefore, some limitations associated with this model are that aged BALB/c mice infected with SARS-CoV-2 developed relatively mild clinical symptoms compared to SARS-CoV-2-infected, hACE2-transgenic mice. Nonetheless, SARS-CoV-2 rapidly adapted in aged BALB/c mice and induced typical pneumonia, which helps to better understand the pathogenesis of SARS-CoV-2 and accelerate the development of countermeasures against COVID-19.

## MATERIALS AND METHODS

### Ethics statement.

All animal experiments were approved by the Research Ethics Committee, Huazhong Agricultural University, Hubei, China (HZAUMO-2016-022) and were performed in accordance with the Guidelines for the Care and Use of Laboratory Animals of the Research Ethics Committee, Huazhong Agricultural University, Hubei, China.

### Virus and cell lines.

The original wild-type SARS-CoV-2 strain (BetaCoV/Wuhan/SARS-CoV-2-WIV04; GISAID accession number EPI_ISL_402124) is referred to as WH-01. It was obtained from the Wuhan Institute of Virology. Viruses recovered after the ninth passage in Vero-E6 cells, referred to as WH-CD (hCoV-19/Wuhan/WH-CD/2020; GISAID accession no. EPI_ISL_1039208), were used for infection. The virus was isolated in Vero-E6 cells (ATCC CRL-1586), cultured in Dulbecco's modified Eagle's medium (DMEM; Invitrogen, Carlsbad, CA, USA) supplemented with 10% fetal bovine serum (FBS), 100 IU/ml penicillin, and 100 μg/ml streptomycin, and incubated at 37°C under a 5% CO_2_ atmosphere. Ten-fold serial dilutions of the virus were titrated in Vero-E6 cells. Three days after inoculation, noticeable cytopathic effects (CPE) were scored, and 50% tissue culture infective doses (TCID_50_) were calculated using the Reed-Muench formula.

### Animal experiments.

Specific pathogen-free (SPF), 12-month-old, male and female BALB/c mice were obtained from Laboratory Animal Services Centre (Huazhong Agricultural University). All experiments were conducted in a biosafety level 3 (BSL-3) core facility at Huazhong Agricultural University. Aged and young mice were anesthetized intraperitoneally (i.p.) with 2.5% avertin (0.02 ml/g body weight) and then inoculated intranasally (i.n.) with WH-CD (1 × 10^5^ TCID_50_). A control group of aged males, aged females, and young males (*n* = 6) was inoculated with DMEM (mock-infected). Survival (*n* = 5) and pathology (*n* = 9) groups comprising aged males, aged females, and young males were inoculated with the virus (infected). Body weight, clinical symptoms, response to external stimuli, and death were assessed daily in the infected animals. Three mice were euthanized on days 3, 5, and 7 via an i.p. injection of pentobarbital (200 mg/kg). Left lungs collected to determine viral load were homogenized in 1 ml of phosphate-buffered saline (PBS). Brain, nasal turbinates, right lungs, liver, heart, spleen, duodenum, and kidneys were fixed in 4% paraformaldehyde for histopathological analysis.

### Plasma cytokine assays.

Blood samples were collected via retro-orbital bleeding from mice under inhaled ether anesthesia. Sera were separated from these samples via centrifugation at 3,000 × *g* for 10 min at 4°C and then stored at −80°C. Serum cytokine levels were measured using mouse Th1/Th2/Th17 Cytokine CBA kits (interleukin-2 [IL-2], IL-4, IL-6, IL-10, IL-17A, TNF-α, and interferon gamma [IFN-γ]) (Becton, Dickinson and Co., Franklin Lakes, NJ, USA) according to manufacturer’s instructions. All serum samples were heat-inactivated by prior incubation at 56°C for 30 min. Briefly, seven bead populations with distinct allophycocyanin (APC)-A fluorescence intensity were coated with capture antibodies (Ab) specific to IL-2, IL-4, IL-6, IFN-γ, TNF-α, IL-17A, and IL-10 proteins, indicated as A1 to A7, respectively. Cytokine concentrations, determined as the fluorescence intensity of phycoerythrin (PE)-conjugated capture Ab, were calculated according to a standard curve of cytokines using FCAP Array software (https://fcap-array.software.informer.com/3.0/). The blank control was assay diluent incubated with the beads and PE-conjugated Ab.

### Determination of SARS-CoV-2 titers.

Vero-E6 cells were seeded in 24-well tissue culture plates overnight at 37°C under 5% CO_2_ atmosphere. Infected lung tissues from euthanized mice were homogenized in DMEM containing piperacillin (Sigma-Aldrich Corp., St. Louis, MO, USA), gentamicin (Invitrogen), and amphotericin B (Sigma-Aldrich Corp., St. Louis, MO, USA) at final concentrations of 0.4, 0.1, and 5 mg/liter, respectively. Duplicate samples were seeded in duplicate wells (100 μl) and incubated at 37°C for 45 min with occasional rocking. Thereafter, 2 ml/well of 0.5% agarose in minimal essential medium (MEM) containing 2% FBS and antibiotics was added to the wells. After 72 h, cells were fixed by immersion in 10% formaldehyde in phosphate-buffered saline (PBS) for 24 h, the agarose plugs were then removed, and the cells were stained with 0.1% crystal violet to visualize PFU. All assays were conducted in the BSL-3 laboratory.

### Plaque reduction neutralization tests.

Neutralizing antibodies in mouse sera were measured using duplicate 50% plaque reduction neutralization tests (PRNT_50_) of Vero cells seeded into 24-well tissue culture plates at the BSL-3 facility. Serum samples were serially diluted and incubated with ∼30 to 40 plaque-forming units (PFU) of SARS-CoV-2 for 1 h at 37°C. The mixtures were then added to Vero E6 cell monolayers and incubated for an additional 1 h at 37°C under 5% CO_2_ atmosphere. The wells were overlaid with 1% agarose in cell culture medium and incubated for 3 days, after which the cells were fixed and stained. Antibody titers were defined as the highest serum dilution that resulted in >90% (PRNT_90_) reduction in the number of plaques.

### RNA isolation and viral load analysis.

Total RNA was extracted from mouse tissues suspended in cold DMEM using QIAamp viral RNA minikits (Qiagen GmbH, Hilden, Germany) and eluted with 30 μl of water. Single-strand cDNA was synthesized from total viral RNA using PrimeScript RT reagent kits (TaKaRa Bio Inc., Kusatsu, Japan). The N gene of SARS-CoV-2 was detected in 2 μl of cDNA using quantitative real-time reverse transcription PCR (qRT-PCR) and quantified using TaqMan Fast Virus 1-Step Master Mix (Thermo Fisher Scientific Inc., Waltham, MA, USA). The primer and probe sequences for the N gene assay were: 5′-TAATCAGACAAGGAACTGATTA-3′ (forward), 5′-CGAAGGTGTGACTTCCATG-3′ (reverse), and 5′-GCAAATTGTGCAATTTGCGG-3′ (probe in 5′-FAM/ZEN/3′-IBFQ format). The predicted amplicon sizes for the N gene assays were 110 bp. All primers and probes were synthesized at a commercial laboratory (Sangon Biotech, Shanghai, China).

### Pathological examination.

Tissues were obtained from euthanized mock- or SARS-CoV-2-infected mice during necropsy. The tissues were fixed in 10% buffered formalin for 72 h, transferred to 70% ethanol, and then washed in PBS for paraffin embedding. The embedded tissues were routinely cut into ∼3- to 4-μm thick sections and then stained with hematoxylin and eosin (H&E) and Masson trichrome for assessment by a veterinary pathologist.

### Immunohistochemistry.

Deparaffinized, rehydrated tissue sections were incubated in antigen retrieval buffer for 15 min at 97°C and endogenous peroxidase was quenched using 3% H_2_O_2_ in methanol for 10 min. Nonspecific protein binding was blocked using 1% normal goat serum and then the sections were incubated with SARS-CoV-2 nucleocapsid rabbit and spike S1 rabbit monoclonal antibodies (1:500) to SARS-CoV-2 (catalog numbers 40143-R019 and 40150-R007, respectively; both from Sino Biological Inc., Beijing, China) at 4°C overnight, followed by horseradish peroxidase (HRP)-labeled goat anti-rabbit IgG secondary antibody (catalog number BM3894; Boster Biological Technology, Wuhan, China). We detected F4/80 (1:100 dilution), CD19 (1:1000 dilution), and IL-6 (1:200 dilution) using rabbit polyclonal antibodies (catalog numbers ab111101, ab245235, and ab208113, respectively; Abcam PLC, Cambridge, UK). The sections were then incubated with a species-specific secondary antibody (Histostain-streptavidin-peroxidase kit, rabbit, number SP-0023, Bioss), developed with 3,3′-diaminobenzine (DAB; Invitrogen), and counterstained with hematoxylin. The sections were examined using a BX-63 microscope (Olympus, Tokyo, Japan). Images were acquired using cellSens software (Olympus). Five random sections were chosen for the intensity quantification. We performed computational analysis of immunohistochemistry (IHC)-stained lung digital images using the Definiens Tissue Studio image analysis software (Version 3.6.1). We programmed the software to segment the images into epithelial and stromal regions for identification and segmentation. The software then automatically extracted summary measures of size, shape, and area from cell membrane and cytoplasmic materials from the IHC images.

### RNA isolation and qPCR.

Total RNAs were extracted by the RNAprep Pure kit (catalog number DP431, Tiangen Biotech, Beijing, China) from the whole lung. For target gene validation, oligo(dT) random hexamers and SuperScript III reverse transcriptase (Life Technologies) were used to synthesize cDNA. qPCR was performed using a SYBR Green Master Mix kit (catalog number RK21203, ABclonal Bio Inc., Wuhan, China). Relative gene expression was analyzed based on the threshold cycle (2^−ΔΔCT^) method with GAPDH as an internal control. At least three independent experiments were analyzed. The primers used for RT-qPCR are listed in Table 1.

**TABLE 1:** Primer sequences and real-time PCR amplification parameters

Gene	Accession no.	Primer (5′–3′)	Product size (bp)	Annealing temp (°C)
GM-CSF	M11848.1	CGGCCTTGGAAGCATGTAGA GCTTGTGTTTCACAGTCCGT	260	60.0
G-CSF	M13926.1	CATGAAGCTAATGGCCCTGC CTGACAGTGACCAGGGGAAC	84	60.0
IL-1β	NM_008361.4	TGCCACCTTTTGACAGTGATG CAAAGGTTTGGAAGCAGCCC	84	60.0
IL-1α	NM_010554.4	CGCTTGAGTCGGCAAAGAAAT CTTCCCGTTGCTTGACGTTG	271	60.0
IL-2	NM_008366.3	ACTGTTGTAAAACTAAAGGGCTCTG GCAGGAGGTACATAGTTATTGAGGG	147	60.0
IL-3	NM_010556.4	GAATGACTCTGCGCTGCCA CTTTAGGTGCTCTGCCTGCT	192	60.0
IL-4	NM_021283.2	CACAGCAACGAAGAACACCA AAGCCCGAAAGAGTCTCTGC	146	60.0
CCL11	NM_011330.3	GCTCACCCAGGCTCCATC TCTCTTTGCCCAACCTGGTC	148	60.0
IL-5	NM_010558.1	CTTCAGAGTCATGAGAAGGATGC CTCCAATGCATAGCTGGTGATTT	203	60.0
IL-6	NM_031168.2	AGTTCCTCTCTGCAAGAGACTTC TTTCCACGATTTCCCAGAGAAC	189	60.0
IL-9	NM_008373.2	GTCTCTCCGTCCCAACTGATGAT CATGGTCGGCTTTTCTGCCTTT	268	60.0
IL-10	NM_010548.2	CCAGCTGGACAACATACTGCTA GAGAAATCGATGACAGCGCC	214	60.0
IL-12B	NM_001303244.1	GGAGGGGTGTAACCAGAAAGGTG TTTTGGGGGACTCTTCCATCCTG	235	60.0
IL-12A	NM_001159424.2	CGTCTACACTGCTGCTGAAAT GTTTCAGTTTTTCTCTGGCCGTC	284	60.0
IL-13	NM_008355.3	CACACAAGACCAGACTCCCC CCAGGGATGGTCTCTCCTCA	288	60.0
IL-17a	NM_010552.3	GGGAGAGCTTCATCTGTGTCTCTG GCGCCAAGGGAGTTAAAGACT	167	60.0
IFN-γ	NM_008337.4	AGGAACTGGCAAAAGGATGGT CTGGTGGACCACTCGGATG	258	60.0
CXCL1	NM_008176.3	ACTCAAGAATGGTCGCGAGG ACTTGGGGACACCTTTTAGCA	89	60.0
CCL3	NM_002983.3	CCAGTTCTCTGCATCACTTGC GAATCTGCCGGGAGGTGTAG	71	60.0
CCL4	NM_013652.2	GCCAGCTGTGGTATTCCTGA TGAACGTGAGGAGCAAGGAC	195	60.0
CCL5	NM_013653.3	CAATCTTGCAGTCGTGTTTGTCA GCCCATTTTCCCAGGACCG	225	60.0
TNF-α	NM_013693.3	AGGCACTCCCCCAAAAGATG CTTGGTGGTTTGCTACGACG	258	60.0
GAPDH	NM_001289726.1	CAGGAGAGTGTTTCCTCGTCC TTCCCATTCTCGGCCTTGAC	222	60.0

### Virus isolation and sequencing.

Infected lung tissues from euthanized mice were homogenized in DMEM and centrifuged at 3,000 rpm for 10 min at 4°C. The supernatant was collected and stored at −80°C for virus load detection and viral isolation as follows. Vero-E6 cell monolayers in 24-well plates were inoculated with 200 μl of supernatants and incubated for 1 h. The supernatant was removed and replaced with fresh DMEM containing 2% FBS and piperacillin (Sigma-Aldrich), gentamicin (Invitrogen), and amphotericin B (Sigma-Aldrich) at final concentrations of 0.4, 0.1, and 5 mg/liter, respectively. The cytopathic effect (CPE) was monitored daily and, after onset, the supernatant was clarified and viral RNA was extracted and purified from 100 μl of culture supernatants using QIAamp viral RNA minikits (Qiagen). Genomic RNA was reverse transcribed using SuperScript IV (Thermo Fisher Scientific, Inc.) according to the manufacturer’s instructions. Next-generation sequencing (NGS) was conducted on an Illumina MiSeq 3000 (Inc., San Diego, CA, USA) and the sequences were analyzed using Geneious 10.2.6 for Windows (https://www.geneious.com/download/). The virus isolated from the infected lungs, referred to as LG (hCoV-19/Wuhan/LG/2020; GISAID accession no. EPI_ISL_1039212), was further confirmed using genome sequencing.

### Statistical analysis.

Data were analyzed with Student’s *t* tests using PRISM 8.0.2 for Windows (GraphPad Software Inc., San Diego, CA, USA). Results in graphs are shown as mean ± standard error of the mean (SEM), unless otherwise stated. All statistics were calculated using GraphPad Prism and display graphs were generated in GraphPad Prism or R. *P* ≤ 0.05 was considered statistically significant.
